# 2269 Day-to-day association between alcohol use and physical activity in university students

**DOI:** 10.1017/cts.2018.59

**Published:** 2018-11-21

**Authors:** Scott Graupensperger, Michael B. Evans

**Affiliations:** Penn State Clinical and Translational Science Institute

## Abstract

OBJECTIVES/SPECIFIC AIMS: The goal of the present study was to advance our understanding of how alcohol use may contribute to physical inactivity among university students by investigating this association at a day-to-day level. METHODS/STUDY POPULATION: In total, 57 university students (Mage=20.27; 54% male) completed daily diary questionnaires using a cellphone application, which prompted them each evening to report minutes of moderate/vigorous physical activity engaged in, and number of alcoholic drinks consumed, as well as intended minutes of physical activity for the following day. Longitudinal mixed-level modeling was used to disentangle within person and between-person effects of alcohol use on physical activity behavior and intentions. Separate models were run to investigate lagged effects of previous day alcohol use. We controlled for sex and age in all models. RESULTS/ANTICIPATED RESULTS: Results indicated that participants’ usual alcohol use (between-person) was not associated with physical activity behavior or intentions. At the within-person level, day-to-day variance in alcohol use was negatively associated with both physical activity behavior (γ=−0.34, *p*=0.003) and intentions to engage in physical activity the following day (γ=−0.70, *p*<0.001). The lagged model indicated that previous day alcohol use negatively predicted PA behavior (γ=−0.33, *p*=0.004). DISCUSSION/SIGNIFICANCE OF IMPACT: Previous studies have largely been constrained to cross-sectional designs, and have surmised that there exists a positive association between alcohol use and physical activity due to trait-level differences between university students. We advance this literature by using ecological momentary assessment to investigate the within-person effects of alcohol use on physical activity at a day-to-day level while controlling for between-person variance. Contrary to existing literature, we found that on days when students consumed relatively more alcohol than they typically report, they: (a) report fewer minutes of physical activity on the same day, (b) plan to engage in relatively less physical activity on the subsequent day, and (c) engage in less physical activity on the subsequent day. By advancing our understanding of how alcohol use may curtail other health behaviors such as physical activity, we inform interventions that aim to target these behaviors in conjunction, or as part of a multiple behavior change intervention.

